# Subthalamic nucleus exclusively evokes dopamine release in the tail of the striatum

**DOI:** 10.1111/jnc.15677

**Published:** 2022-08-04

**Authors:** Kathryn L. Todd, Janusz Lipski, Peter S. Freestone

**Affiliations:** ^1^ Faculty of Medical and Health Sciences University of Auckland Auckland New Zealand

**Keywords:** basal ganglia, dopamine, electrochemistry, substantia nigra pars lateralis, subthalamic nucleus, tail striatum

## Abstract

A distinct population of dopamine neurons in the substantia nigra pars lateralis (SNL) has a unique projection to the most caudolateral (tail) region of the striatum. Here, using two electrochemical techniques to measure basal dopamine and electrically evoked dopamine release in anesthetized rats, we characterized this pathway, and compared it with the ‘classic’ nigrostriatal pathway from neighboring substantia nigra pars compacta (SNc) dopamine neurons to the dorsolateral striatum. We found that the tail striatum constitutes a distinct dopamine domain compared with the dorsolateral striatum, with consistently lower basal and evoked dopamine, and diverse dopamine release kinetics. Importantly, electrical stimulation of the SNL and SNc evoked dopamine release in entirely separate striatal regions; the tail and dorsolateral striatum, respectively. Furthermore, we showed that stimulation of the subthalamic nucleus (STN) evoked dopamine release exclusively in the tail striatum, likely via the SNL, consistent with previous anatomical evidence of STN afferents to SNL dopamine neurons. Our work identifies the STN as an important modulator of dopamine release in a novel dopamine pathway to the tail striatum, largely independent of the classic nigrostriatal pathway, which necessitates a revision of the basal ganglia circuitry with the STN positioned as a central integrator of striatal information.

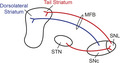

## Graphical abstract

We showed that neurons in the substantia nigra pars lateralis (SNL) constitute a dopamine pathway projecting to the tail of the striatum, a dopamine domain distinct to the dorsolateral striatum which receives input from the substantia nigra pars compacta (SNc). Furthermore, the subthalamic nucleus (STN) exclusively modulates dopamine release in the tail striatum, likely via the SNL. Our work necessitates a revision of the basal ganglia circuitry with the STN positioned as a central integrator of information from both the dorsolateral (motor) and tail (sensory) striatum.

**Abbreviations**
DAT: dopamine transporter
DLS: dorsolateral striatum
FSCV: fast‐scan cyclic voltammetry
FSCAV: fast‐scan controlled‐adsorption voltammetry
MFB: medial forebrain bundle
SNc: substantia nigra pars compacta
SNL: substantia nigra pars lateralis
SNr: substantia nigra pars reticulata
STN: subthalamic nucleus
VGlut2: vesicular glutamate transporter 2
VTA: ventral tegmental area

## 1 INTRODUCTION

Dopamine release in the striatum of the basal ganglia is a fundamental process that underlies many core brain functions including motor control, motivation, and reinforcement learning (Albin et al., 1989; McGregor & Nelson, 2019; Schultz, 2007; Wise, 2004). Indeed, a substantial loss of dopamine‐producing neurons in the substantia nigra pars compacta (SNc) and/or abnormal dopamine transmission in the striatum is the key feature of Parkinson's disease and other neurological disorders such as schizophrenia and ADHD (Blandini et al., 2000; Carlsson, 1977; DeLong, 1990; Miller et al., 2012). A precise understanding of dopamine transmission throughout the striatum, as well as the mechanisms of modulating dopamine are key to advancing our understanding of the pathophysiology of these disorders and their treatments.

Midbrain dopamine neurons are anatomically unique, having limited collateralization and mostly targeting a single region of the brain (Matsuda et al., 2009; Moore & Bloom, 1978), indicating that different populations can be defined by their targets. The intensely studied SNc and ventral tegmental area (VTA) dopamine neurons project via the medial forebrain bundle (MFB) to distinct domains of the striatum; the dorsolateral (sensorimotor) domain and ventral (limbic) domain (nucleus accumbens), respectively (de Jong et al., 2022; Menegas et al., 2015). Anatomical studies in mice have identified an additional dopamine pathway from the most lateral part of the substantia nigra (substantia nigra pars lateralis; SNL) to the most caudal part of the striatum, the tail of the striatum (‘tail striatum’ from here on; Menegas et al., 2015, 2018). This dopamine pathway has also been identified in rats (Jiang & Kim, 2018) and is conserved in primates; in monkeys (Kim et al., 2014) and humans (Zhang et al., 2017) dopamine neurons in the caudal dorsolateral SNc project to the tail of the caudate nucleus, which are areas homologous to the SNL and tail striatum, respectively, in rodents (Jiang & Kim, 2018). In addition to projection‐based differentiation of SNL and SNc dopamine neurons, molecular diversity of these midbrain neurons has been described. Specifically, at least in rodents, SNL dopamine neurons, unlike SNc dopamine neurons, express the vesicular glutamate transporter 2 (VGlut2), have high calbindin expression, low expression of the dopamine transporter (DAT), and lack D_2_ autoreceptors (Fu et al., 2012; Poulin et al., 2018; Wang et al., 2019). Together, this projection and molecular diversity of SNL and SNc dopamine neurons raises the question; are the SNL and SNc capable of independent dopamine release in the tail and dorsolateral striatum, and are there differences in the kinetics of dopamine release between their respective striatal targets?

Despite the striatum extending extensively along the rostro‐caudal axis, much of the current knowledge about striatal function comes from the most rostral (medial‐lateral and ventral) regions. Interestingly, recent findings have highlighted the tail striatum as an additional functional domain of the striatum (see Valjent & Gangarossa, 2021 for review) involved in processing sensory information from auditory and visual cortices (Hunnicutt et al., 2016). However, a direct comparison of dopamine transmission in the tail striatum compared with the rostral striatum is yet to be done.

Of further interest is evidence that SNL dopamine neurons, unlike SNc dopamine neurons, receive relatively little input from the ventral striatum, instead receiving input mainly from the subthalamic nucleus (STN; Menegas et al., 2015). Knowledge of how the STN modulates dopamine release in the tail striatum, via the SNL, is important not only for more complete elucidation of basal ganglia function, but also better understanding of the pathophysiology and treatments of Parkinson's disease given the hyperactivity of this nucleus (Ammari et al., 2010) and success of STN deep brain stimulation in alleviating symptoms in patients (Gill et al., 2011; Okun, 2012).

In this study, we applied conventional fast‐scan cyclic voltammetry (FSCV) and fast‐scan controlled‐adsorption voltammetry (FSCAV) to make a robust comparison of evoked and basal dopamine between the tail and dorsolateral striatum and determine what role the STN has in modulating dopamine release in these two striatal regions.

## 2 MATERIALS AND METHODS

### 2.1 Animals

In vivo experiments were conducted on anesthetized male Wistar rats (7–8 weeks old; 270–310 g). Prior to experiments, rats were housed in cages in a humidity and temperature‐controlled environment with a 12‐h dark/12‐h light cycle and unrestricted access to food and water. All experimental procedures were conducted with approval from the Animal Ethics Committee of the University of Auckland (AEC 001748), in accordance with the New Zealand Government Animal Welfare Act.

### 2.2 Stereotaxic surgery

**FIGURE 1:**
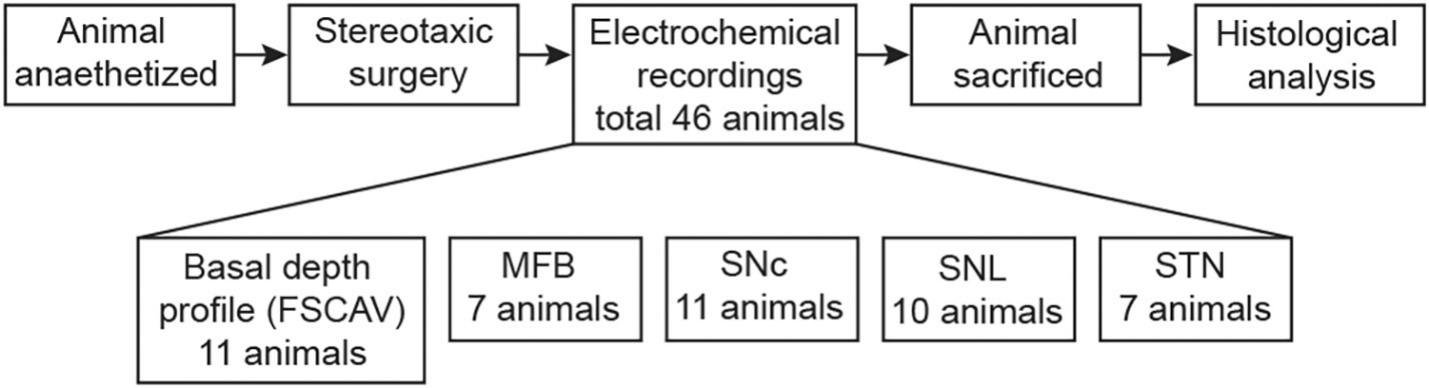
Experimental procedure. Stereotaxic surgery was performed under urethane anesthesia and electrodes were lowered into the relevant brain regions. Electrochemical recordings were conducted in one or both hemispheres. For FSCV recordings, stimulation site (MFB: medial forebrain bundle, SNc: substantia nigra pars compacta, SNL: substantia nigra pars lateralis, STN: subthalamic nucleus) was determined arbitrarily. Basal dopamine measurements (FSCAV) were taken across all experimental groups. Dorsolateral and tail striatum were recorded from sequentially (same hemisphere) in a random order. After experiments, animals were sacrificed by decapitation and brain tissue was stored in fixative for 48 hrs prior to histological validation of stimulation site (data not shown).

Rats were anesthetized with urethane (1.6 g/kg, i.p, 60% then 40% doses 20 min apart; dissolved in sterile 0.9% NaCl; Sigma Aldrich) and, after reaching the surgical plane of anesthesia, were secured in a stereotaxic frame (Model 942; Kopf). Urethane was chosen as the appropriate anesthetic due to its use in other studies involving the electrochemical detection of dopamine (Covey & Garris, 2009; Kuhr et al., 1987; Lloyd et al., 2022). Following subcutaneous marcaine anesthesia (Bupivacaine hydrochloride; 0.2 mg/kg; Multichem NZ) to minimize pain at the incision site, bilateral craniotomies were performed over the relevant brain regions. A Ag/AgCl reference electrode was inserted subcutaneously at the base of the neck. Body temperature was maintained at 36°C (homeothermic monitoring system; Harvard Apparatus), and heart rate (350–450/min) and O_2_ saturation (~99%) were continuously monitored (MouseStat Jr. Rodent Pulse Oximeter; Kent Scientific). Regular subcutaneous injections of sterile 0.9% NaCl were administered every 1–2 hrs (200 μl). Experiments were performed from about 8 a.m. to 9 p.m., with animals anesthetized at 8–10 a.m. At the end of experiments, rats were sacrificed by decapitation and the brain was removed. A small block of tissue containing the relevant brain structures was cut and placed in fixative (4% paraformaldehyde) for 48 hrs prior to washing and storage in phosphate‐buffered saline. The experimental procedure is outlined in Figure 1.

### 2.3 Electrochemistry

Carbon‐fiber microelectrodes were manufactured as described previously (Burrell et al., 2015; Yee et al., 2019). Briefly, a carbon fiber (7 μm diameter; Goodfellow Cambridge Ltd) was threaded through a pulled borosilicate glass pipette (3.0 mm o.d., 1.62 mm i.d.; Harvard Apparatus) after breaking the tip to a diameter of 10–15 μm. The tip was sealed and electrically insulated by back injection of epoxy resin (Epoxylite; Epoxylite Corp) and cured overnight in an oven (120°C). A copper wire soldered to a gold‐plated socket was inserted into the glass and secured with carbon‐based wire glue (Anders Products). The carbon fiber protruding from the glass tip was trimmed to a length of 60–100 μm. To improve selectivity and sensitivity for detection of dopamine, carbon‐fiber microelectrodes were coated with a Nafion and poly(3,4‐ethylenedioxythiophene) (PEDOT) composite polymer by electrodeposition (Vreeland et al., 2015). Microelectrodes were submerged in a deposition solution containing EDOT (200 μM; 3,4‐ethylenedioxythiophene; Sigma Aldrich; Cat# 483028) and Nafion (1%; Sigma Aldrich; Cat# 274704) dissolved in acetonitrile (HPLC grade; Sigma Aldrich). Deposition was performed by applying a triangle waveform from +1.5 V to −0.8 V at 100 mV/s for 15 cycles. Carbon‐fiber microelectrodes were calibrated prior to every experiment in a beaker containing Tris‐buffered ACSF (mM: 127 NaCl, 3 KCl, 2 CaCl_2_, 1.25 NaH_2_PO_4_, 2 MgSO_4_, 10 Tris–HCl; pH 7.4). Calibration curves were obtained by adding aliquots of concentrated dopamine hydrochloride solution (100 μM in distilled water; Sigma Aldrich; Cat# H8502) in 100–250 nM steps.

#### 2.3.1 Fast‐scan cyclic voltammetry (FSCV)

FSCV was performed to measure evoked dopamine release in vivo. A carbon‐fiber microelectrode was lowered into either the dorsolateral (AP 0, ML ‐3.2, DV ‐5.0, relative to bregma) or tail striatum (AP ‐1.8, ML ‐3.2, DV ‐3.5). Electrode potential was controlled with customized WCCV software (WCCV 3.0; Knowmad Technologies) interfaced with a potentiostat (Chem‐Clamp; Dagan Corp) and a PCI Data Acquisition card (PCI‐6321; National Instruments). For FSCV, the electrode potential was scanned as a triangular waveform (−0.4 V to +1.3 V; 400 V/s) at 10 Hz. The resultant current was filtered (10 kHz) and recorded using the potentiostat and WCCV software. After insertion of the carbon‐fiber microelectrode into the brain, recordings started following a short period (5–10 min) of scanning to allow electrode stabilization.

#### 2.3.2 Fast‐scan controlled adsorption voltammetry (FSCAV)

FSCAV was performed to measure absolute (basal) levels of dopamine concentration. Voltage commands applied to the microelectrode alternated between a triangular waveform (−0.4 V to +1.3 V; 1200 V/s) repeated at 100 Hz (10 s) and a constant DC potential (−0.4 V; 10 s). This pause in scanning allowed for maximal adsorption of dopamine to the carbon‐fiber surface (Atcherley et al., 2013). Switching between voltage commands was achieved with a CMOS precision analog switch (ADG419; Analog Devices), gated by a TTL pulse from the PCI‐6321 acquisition card. Live monitoring of basal dopamine during experiments was achieved using custom software (Live Electrochemistry; Peter S. Freestone).

### 2.4 Electrical stimulation

A twisted pair bipolar electrode (125 μm diameter; MS303/8‐B/SPC; PlasticsOne) was lowered into the brain (1 mm/min) to above either the MFB (AP ‐4.4, ML −1.4), SNc (AP ‐5.2, ML ‐2.0), SNL (AP ‐6.0, ML −3.0) or STN (AP ‐3.6, ML −2.5). The electrode was then slowly advanced while stimulating every 50–200 μm until a maximal evoked response was observed. Stimulation sites were confirmed following histological analysis post‐experiment. Bi‐phasic constant current stimuli (60 Hz, 120 pulses, 300 μA, 2 ms each phase) were generated using a stimulus isolator (DS4; Digitimer) with timing parameters controlled by WCCV software. The electrical stimuli were applied so that there was no overlap with the FSCV scans to avoid noise caused by the stimulus artifact.

### 2.5 Electrochemical analysis

Electrochemical data were analyzed using WCCV software and Excel (Microsoft). For analysis of FSCV recordings, the current at the dopamine oxidation peak was converted to dopamine concentration using calibration data (fitted with a second‐order polynomial). An increase in dopamine concentration was only considered significant if the cyclic voltammogram had an oxidation peak between 0.5 and 0.6 V at least 1.5 times the noise level, otherwise they were excluded from the FSCV data. The reproducibility of evoked responses per stimulation site (% of total brain hemispheres stimulated) is reported in the figures. Values of interest including amplitude and time to peak were calculated using Excel. Differentiation (first derivative) of the dopamine response was performed in OriginPro 2021 (OriginLab Corporation) to determine profiles of velocity of dopamine release. For FSCAV recordings, a segment (between +0.4 V and + 0.9 V) of the 10th scan after the pause was integrated using WCCV software. This scan was chosen because of its high selectivity for dopamine over its metabolites and other electrochemically active species (Atcherley et al., 2013; Burrell et al., 2015). The integrated area (charge, pC) was converted to dopamine concentration using calibration data (fitted with a second order polynomial) and plotted against time. Absolute basal dopamine concentration was calculated from the average concentration over a 5 min stable period.

### 2.6 Statistical analysis

A total of 46 Wistar rats were used in this study. No randomization or blinding was performed in this study, and brain regions (recording and stimulation sites) were assessed in an arbitrary order. Sample size numbers represent the number of brain hemispheres. No sample size calculation was performed prior to the study and this study was exploratory. Post‐hoc power analysis calculated a minimum sample size of 3 (σ = 24 nM, precision = 20 nM, α = 0.05), validating the sample sizes used in this study (*n* ≥ 4). Statistical tests were performed in SPSS (IBM) and graphical presentation was performed using OriginPro 2021. Statistical tests included two‐tailed paired and independent samples *t*‐tests. Normality was tested using Shapiro–Wilk's test and homogeneity of variance was tested using Levene's test. If these assumptions were violated, an equivalent non‐parametric test (Mann–Whitney *U* test) was performed. Differences were considered statistically significant if *p* < 0.05. No test for outliers was conducted and no outliers were removed. Data are presented as mean ± SEM in the text, and in figures as either box and whisker plots depicting the minimum, first quartile, mean, third quartile and maximum or as bars representing the mean ± SEM. This study was not pre‐registered.

## 3 RESULTS

### 3.1 Basal dopamine profiles reveal distinct dorsolateral and tail striatum domains

**FIGURE 2:**
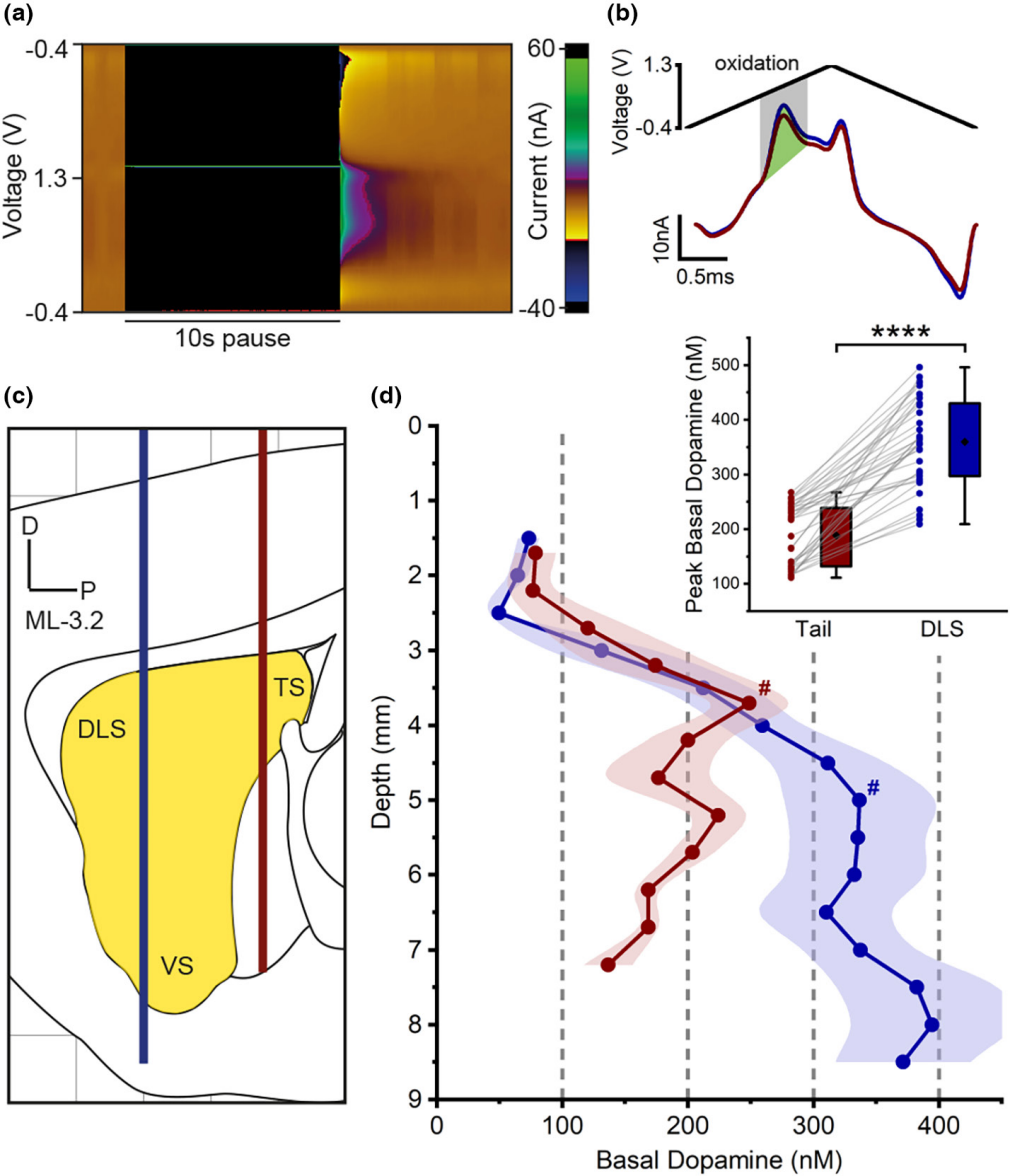
Basal dopamine profiles reveal distinct dorsolateral and tail striatum domains. (a) Color plot showing oxidation and reduction currents of dopamine adsorbed to the carbon‐fiber microelectrode after the 10 s pause in fast‐scan controlled adsorption voltammetry (FSCAV). (b) Voltammograms (10th scan after pause) showing dopamine oxidation current recorded in the tail (red) and dorsolateral (blue) striatum in response to the cyclic voltage ramp (top). Green area represents the integral of current (charge, pC) used to calculate dopamine concentration. (c) Representative sagittal section (ML −3.2) indicating rostral (AP 0; blue) and caudal (AP ‐1.8; red) recording tracts. DLS, dorsolateral striatum; TS, tail striatum; VS, ventral striatum. (d) Distinct depth profiles of basal dopamine in the rostral (blue; *n* = 6) and caudal (red; *n* = 7) recording tracts. Inset: Peak basal dopamine (#) was consistently higher in the dorsolateral striatum (DV ‐5.0) compared with tail striatum (DV ‐3.5) in all experiments (*n* = 35). Connecting lines indicate paired measurements (same hemisphere, sequentially measured). *n* = number of hemispheres. ****p < 0.0001.

To determine if basal dopamine differs between the tail and dorsolateral striatum, FSCAV (Atcherley et al., 2013; Burrell et al., 2015) was used to make accurate, stable recordings of basal dopamine in anesthetized rats (Figure 2).

Basal dopamine was measured at multiple depths (at 500 μm intervals) within the same tract at caudal and rostral locations (Figure 2c). In the cortex immediately above the striatum, basal dopamine was comparable in caudal (77 ± 12 nM; *n* = 7) and rostral tracts (73 ± 9 nM; *n* = 6; *t*[11] = 0.2, *p* = 0.8, independent t‐test). Going deeper, two distinct peaks of dopamine concentration were seen in the caudal tract (Figure 2d) corresponding to the tail striatum (249 ± 31 nM at 3.7 mm deep) and the globus pallidus (GP; 224 ± 23 nM at 5.2 mm deep), which is known to receive dopaminergic innervation from collaterals of the nigrostriatal pathway (Hernández et al., 2007; Lindvall, 1979). In the rostral tract, two broader peaks were observed corresponding to the dorsolateral striatum (337 ± 60 nM at 5.0 mm deep) and ventral striatum (395 ± 65 nM at 8.0 mm deep).

Further recordings from the depth of the first peak in each tract revealed that basal dopamine was consistently lower in the tail striatum compared with the dorsolateral striatum (tail, 188 ± 9 nM; dorsolateral, 360 ± 13 nM; *t*[34] = 13.1, *p* < 0.0001, paired t‐test; *n* = 35; Figure 2d). In both regions, the cyclic voltammogram peak oxidation current occurred at a voltage consistent with the oxidation of dopamine (tail, 0.664 ± 0.003 V; dorsolateral, 0.666 ± 0.002 V; Figure 2b).

### 3.2 MFB stimulation evokes dopamine release in both dorsolateral and tail striatum

**FIGURE 3:**
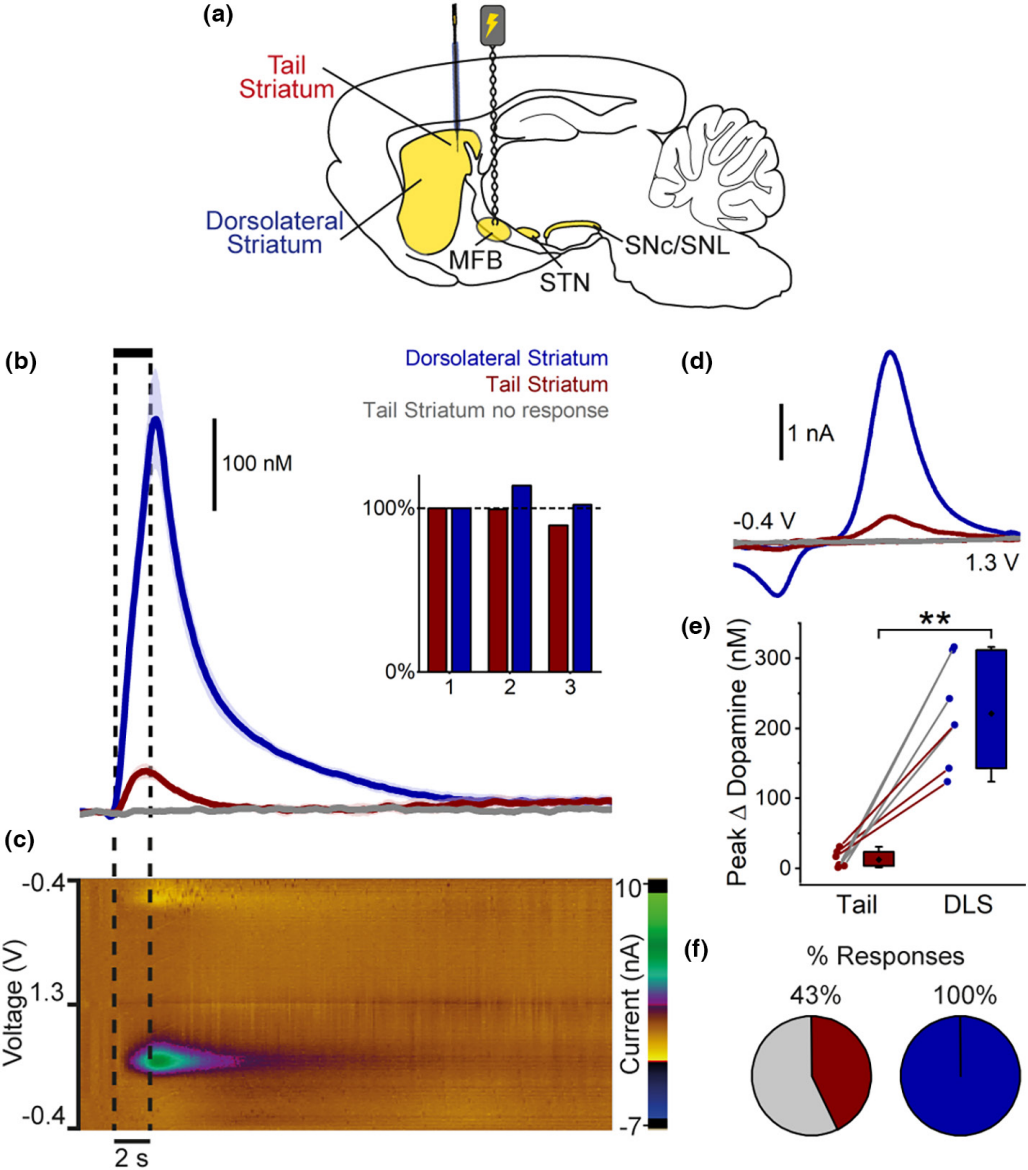
MFB stimulation evokes dopamine release in both dorsolateral and tail striatum. (a) Schematic (sagittal section) showing carbon‐fiber microelectrode recording locations in the dorsolateral (DV ‐5.0) and tail (DV ‐3.5) striatum, and stimulation sites in the medial forebrain bundle (MFB), subthalamic nucleus (STN), substantia nigra pars compacta (SNc), and pars lateralis (SNL). Results for STN, SNc and SNL stimulation shown in Figure 4. (b) MFB stimulation (60 Hz, 120 pulses, 300 μA, 2 ms each phase; vertical lines) evoked pronounced dopamine release in the dorsolateral striatum (DLS; blue; *n* = 7), while recording in the tail striatum revealed much smaller release (red; *n* = 3), or no detectable release at all (gray; *n* = 4). *n* = number of hemispheres. Inset: Consistency (% of first response) of responses across repeated stimulations at 5 min intervals. (c) Color plot showing oxidation and reduction currents following MFB stimulation (vertical lines) recorded in the dorsolateral striatum. (d) Corresponding average cyclic voltammograms showing oxidation and reduction currents consistent with dopamine. (e) Peak dopamine release following MFB stimulation was significantly higher in the DLS compared to the tail striatum. Connecting lines indicate paired measurements (same hemisphere, sequentially measured). Gray connecting lines indicate pairs of recordings in which no dopamine release was detected in the tail striatum (see B). **p < 0.01. (f) MFB stimulation faithfully evoked dopamine release in the dorsolateral striatum (7 from 7 hemispheres; blue), but less reliably in the tail striatum (3 from 7 hemispheres; red).

Striatal dopamine release has traditionally been studied using electrical stimulation of the MFB. To determine if tail striatum‐projections are also carried via the MFB, FSCV recordings were conducted to measure evoked dopamine release in the tail and dorsolateral striatum sequentially (randomized order) following electrical stimulation of the MFB (60 Hz, 120 pulses, 300 μA, 2 ms each phase; Figure 3a).

Consistent with previous studies (Covey & Garris, 2009; Kuhr et al., 1987), MFB stimulation (DV 9.0 ± 0.1) evoked large and robust dopamine release in the ipsilateral dorsolateral striatum (Figure 3b,c). Here we show, for the first time, that MFB stimulation also evoked dopamine release in the tail striatum. This was only detectable (see methods) in 3 of the 7 experiments, while release in the dorsolateral striatum was observed in all experiments (Figure 3f). MFB‐evoked dopamine release in the tail striatum had a significantly smaller amplitude than in the dorsolateral striatum (tail, 24 ± 4 nM, *n* = 3; dorsolateral, 221 ± 28 nM, *n* = 7; *t*[8] = 4.4, *p* = 0.002; Figure 3e) and faster time to peak (tail, 1.7 ± 0.1 s; dorsolateral, 2.3 ± 0.1 s; *t*[8] = 5.2, *p* = 0.001; all independent *t*‐tests). Repeated stimulation (at 5 min intervals) evoked consistent dopamine release with minimal variation in amplitude (Figure 3b inset). Cyclic voltammogram peak oxidation current occurred at a voltage consistent with the detection of dopamine (tail, 0.514 ± 0.005 V; dorsolateral, 0.529 ± 0.003 V; Figure 3d).

To investigate the low reproducibility of MFB‐evoked dopamine release in the tail striatum, histological analysis was conducted to confirm the exact location of MFB stimulation. Interestingly, dopamine release in the tail striatum was only evoked when stimulating at a more caudal site along the MFB (data not shown).

### 3.3 SNL and STN stimulation exclusively evoke dopamine release in the tail striatum

To further evaluate the novel dopamine pathway, dopamine release was recorded in both the tail and dorsolateral striatum sequentially (randomized order) in response to electrical stimulation of the SNL and STN and compared with SNc‐evoked dopamine release.

**FIGURE 4:**
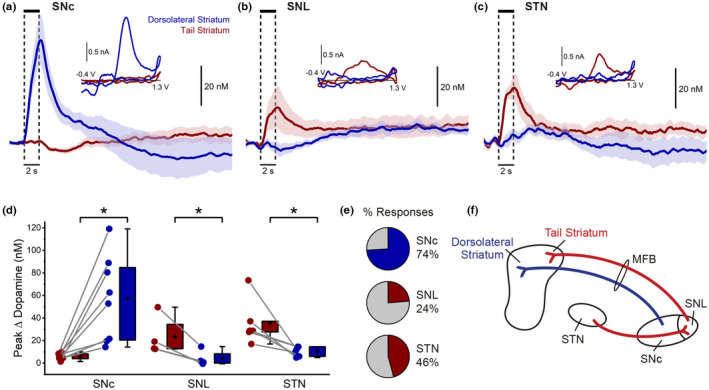
SNL and STN stimulation exclusively evoke dopamine release in the tail striatum. (a–c) Stimulation of the substantia nigra pars compacta (a, SNc; *n* = 8) evoked dopamine release in the dorsolateral striatum (blue), with no release detected in the tail striatum (red). Conversely, stimulation of the substantia nigra pars lateralis (b, SNL; *n* = 4) or subthalamic nucleus (c, STN; *n* = 6) evoked dopamine release in the tail striatum, with no release detected in the dorsolateral striatum. *n* = number of hemispheres. Insets: Corresponding average cyclic voltammograms. (d) Comparison of peak dopamine release in the tail (red) and dorsolateral (blue) striatum. Connecting lines indicate paired measurements (same hemisphere, sequentially measured). *p < 0.05. (e) Stimulation of the SNc more reliably evoked dopamine release in the dorsolateral striatum compared with release in the tail striatum following SNL and STN stimulation. (f) Proposed schematic of distinct dopamine pathways from the SNc and SNL to the distinct dopamine domains in the dorsolateral and tail striatum, respectively, and selective activation of the SNL by the STN.

As expected, SNc stimulation (DV 8.4 ± 0.1) evoked dopamine release in the dorsolateral striatum with an amplitude of 57 ± 13 nM (*n* = 8; Figure 4a), as indicated by a distinct oxidation peak at 0.55 ± 0.01 V. Conversely, no dopamine release in the tail striatum was detected.

In complete contrast, SNL stimulation (DV 7.4 ± 0.2) evoked small dopamine release in the tail striatum (24 ± 9.0 nM; oxidation peak at 0.57 ± 0.02 V; *n* = 4), but no dopamine release in the dorsolateral striatum (Figure 4b). These findings confirm that the SNc and SNL are distinct populations of dopamine neurons which differentially innervate the dorsolateral and tail striatum, respectively.

Stimulation of the STN (DV 8.3 ± 0.1) evoked small dopamine release in the tail striatum (36 ± 8 nM; oxidation peak at 0.56 ± 0.02 V; *n* = 6), consistent with anatomical evidence of a significant STN to SNL projection (Menegas et al., 2015). Conversely, there was no detectable dopamine release in the dorsolateral striatum (Figure 4c), thus showing an identical pattern of dopamine release in the striatum as SNL stimulation (Figure 4d). Dopamine release in the dorsolateral striatum (SNc stimulation) was more reliably evoked than in the tail striatum (SNL and STN stimulation; Figure 4e). These results reveal the preferential modulatory role of the STN on SNL dopamine neurons and dopamine release in the tail striatum (Figure 4f). The absence of evoked release in the dorsolateral striatum confirms that our STN stimulation is specific and not inadvertently activating the MFB which passes close by.

### 3.4 Distinct dopamine release kinetics in the tail and dorsolateral striatum

**FIGURE 5:**
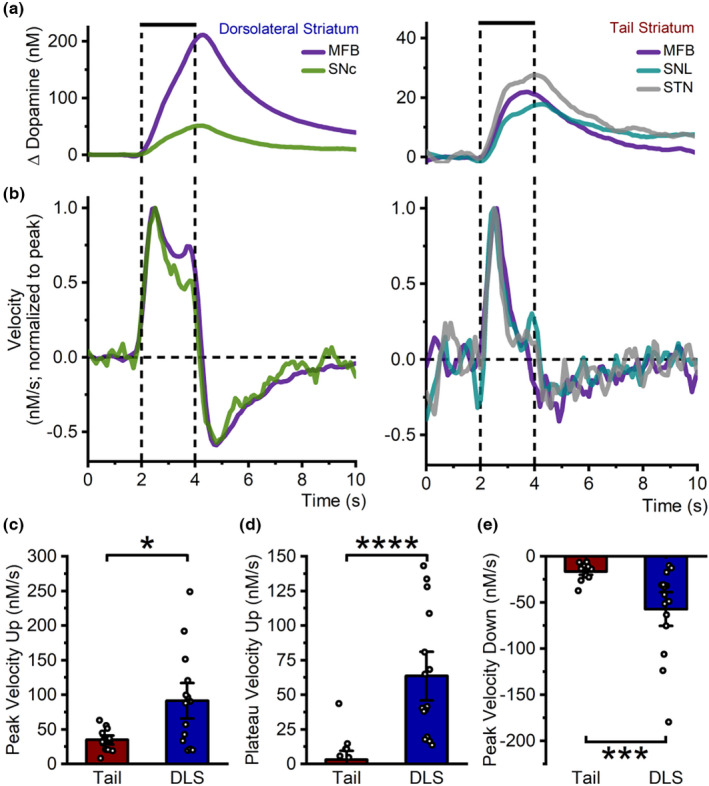
Distinct dopamine release kinetics in the tail and dorsolateral striatum. (a) Dopamine release in the dorsolateral (left) and tail (right) striatum had distinct profiles, independent of stimulation site (MFB: medial forebrain bundle, DLS *n* = 7, tail *n* = 3; SNc: substantia nigra pars compacta, *n* = 8; SNL: substantia nigra pars lateralis, *n* = 4; STN: subthalamic nucleus, *n* = 6; stimulation 60 Hz, 120 pulses, 300 μA, 2 ms each phase, vertical lines). (b) Velocity of dopamine release (first derivative, normalized to peak upward velocity) had distinct profiles between the dorsolateral (left) and tail (right) striatum with dopamine release in the dorsolateral striatum having obvious sustained release for the duration of the stimulation, while release in the tail striatum declined quickly after an initial phase despite ongoing stimulation. (c–e) Summary of grouped data showing that the tail striatum (red; *n* = 13) had a slower peak (c) and plateau (d) upward velocity as well as slower peak downward velocity (e), compared with the dorsolateral striatum (DLS, blue; *n* = 15). *n* = number of hemispheres. *p < 0.05, ***p < 0.001, ****p < 0.0001.

Investigation of dopamine release and clearance kinetics can reveal information about the modulation of dopamine transmission, and how it differs between the two striatal domains. The velocity of dopamine release in the tail and dorsolateral striatum was determined by differentiating (first derivative; Everett et al., 2022) the evoked responses which was then grouped by striatal recording location (Figure 5a). In both striatal regions, dopamine release occurred in two phases. In the dorsolateral striatum there was a fast initial phase of release (peak upward velocity, 91 ± 17 nM/s; *n* = 15), which was then sustained at a slower rate (plateau upward velocity, 64 ± 12 nM/s) for the duration of the stimulation (also seen in other studies; Covey & Garris, 2009; Min et al., 2016). Conversely, in the tail striatum the velocity of dopamine release declined quickly after a slower initial phase (peak upward velocity, 35 ± 4 nM/s; *n* = 13) to near‐zero (plateau upward velocity, 3 ± 4 nM/s) in the second phase despite ongoing stimulation (Figure 5b). The peak and plateau upward velocities of dopamine release were significantly slower in the tail striatum compared with the dorsolateral striatum (peak, U = 43, *p* = 0.011; Figure 5c; plateau, U = 9, *p* < 0.0001, Mann–Whitney U test; Figure 5d). To analyze dopamine clearance, we used the peak downward velocity. This was significantly slower in the tail striatum (−16 ± 2 nM/s; *n* = 13) compared with the dorsolateral striatum (−57 ± 12 nM/s; *n* = 15; U = 28, *p* = 0.0008, Mann–Whitney U test; Figure 5e). Amplitude and kinetic values were found to be linearly correlated; peak upward velocity (*R* ^2^ = 0.9), plateau upward velocity (*R* ^2^ = 0.86), and peak downward velocity (*R* ^2^ = 0.88).

## 4 DISCUSSION

This study provides the first functional characterization of the novel dopamine pathway from a separate population of dopamine neurons in the SNL to the tail striatum in rats using electrochemical detection of dopamine. We show that the tail striatum is a distinct dopamine domain having unique basal and evoked dopamine characteristics compared with the dorsolateral striatum. Furthermore, dopamine release in the tail and dorsolateral striatum was differentially evoked by stimulation of the SNL and SNc, respectively. Finally, we found that the STN exclusively evoked SNL‐mediated dopamine release in the tail striatum, and not SNc‐mediated release in the dorsolateral striatum.

### 4.1 The tail and dorsolateral striatum are distinct dopamine domains

In this study we found that basal dopamine was lower in the tail striatum and had distinct dopamine release kinetics compared with the dorsolateral striatum, suggesting these are indeed two distinct striatal dopamine domains.

We employed an electrochemical technique (FSCAV), based on FSCV, to describe high resolution depth profiles of basal dopamine in rostral and caudal striatal regions with sub‐millimeter resolution. These profiles closely matched the underlying anatomically and functionally defined regions of the striatum (Floresco, 2015; Hunnicutt et al., 2016; Voorn et al., 2004); dorsal and ventral domains rostrally corresponded to the dorsolateral striatum and nucleus accumbens, respectively, and the caudal domain to the tail striatum. Basal dopamine concentration in the dorsolateral striatum measured here is consistent with previous observations using the same technique (FSCAV; DiCarlo et al., 2019; Lloyd et al., 2022). Notably, these values are 10–40‐fold greater than those previously obtained using microdialysis (Gu et al., 2015; Shou et al., 2006), likely explained by the significant tissue damage associated with the microdialysis probe (Ø > 200 μm) leading to underestimation of dopamine concentration (Bungay et al., 2003; Yang et al., 1998).

Both basal dopamine and evoked release were consistently lower in the tail striatum compared with the dorsolateral striatum. Furthermore, measures of dopamine release kinetics including peak upward velocity, plateau upward velocity, and peak downward velocity were significantly slower in the tail striatum compared with the dorsolateral striatum. Dopamine release kinetics were remarkably similar within each dopamine pathway irrespective of stimulation site, and were instead determined by the striatal region recordings were made in. Several possible reasons could explain all these differences between the tail and dorsolateral striatum. Firstly, expression of tyrosine hydroxylase is lower in the tail striatum (Miyamoto et al., 2019; Ogata et al., 2022). This finding, albeit in mouse, could be explained by a comparatively lower density of dopamine terminals in the tail striatum, which would lead to less spontaneous dopamine release and subsequent lower basal concentration (Liu et al., 2021; Rice et al., 2011) as well as smaller evoked dopamine release. Indeed, unlike the dorsolateral striatum which receives input from densely packed dopamine neurons in the SNc that branch extensively (Matsuda et al., 2009), the tail striatum is innervated by a more sparsely populated cluster of dopamine neurons in the SNL (Fu et al., 2012; González‐Hernández & Rodríguez, 2000). Furthermore, unlike the relatively homogenous dorsolateral striatum, studies have shown that the tail striatum comprises of multiple subdivisions, notably two broad ventral regions lacking either the D_1_ or D_2_ dopamine receptors (Gangarossa et al., 2013; Miyamoto et al., 2019; Ogata et al., 2022). These subdivisions also have different compositions of GABAergic and cholinergic interneurons, which, through cortico‐striatal input, can modulate basal dopamine (Abudukeyoumu et al., 2019; Lopes et al., 2019; Roberts et al., 2021). This study did not discriminate between all subregions within the tail striatum, with recordings primarily made in the dorsal aspect of the tail striatum. Future studies are needed to investigate dopamine transmission in the ventral D_1_ and D_2_‐poor subregions of the tail striatum. The striatum is also spatially organized into compartments known as striosomes (patches) and matrix (Desban et al., 1993) and recent evidence has shown that these compartments correlate to fast and slow kinetics of dopamine release, respectively, with differences observed between the dorsolateral and dorsomedial striatum (Jaquins‐Gerstl et al., 2021). Differences in the patch/matrix organization of the tail striatum, of which little is known, compared with the dorsolateral striatum could also contribute to the differences in dopamine release kinetics observed here. Finally, the distinct dopamine release kinetics in the tail and dorsolateral striatum suggests that dopamine uptake and recycling mechanisms are different between the two striatal regions. Potential differences, including DAT efficiency (Cragg et al., 2002; Jones et al., 1995) and D_2_ autoreceptor regulation (Davidson & Stamford, 1993; Trout & Kruk, 1992), do exist between the dorsal and ventral striatum (nucleus accumbens; Calipari et al., 2012). Further studies using pharmacological intervention or a DAT‐knockout model (Lloyd et al., 2022) would greatly advance our understanding of the different dopamine transmission mechanisms in the tail and dorsolateral striatum.

### 4.2 The tail and dorsolateral striatum are innervated by distinct dopamine pathways

Anatomical studies have recently described a midbrain projection to the tail striatum from a distinct population of dopamine neurons residing in the SNL (Jiang & Kim, 2018; Menegas et al., 2015, 2018), molecularly distinct from adjacent SNc dopamine neurons (Poulin et al., 2018, 2020). We provide the first functional evidence of this dopamine pathway by directly observing evoked dopamine release exclusively in the tail striatum following electrical stimulation of the SNL. In total contrast to the better studied projection of neighboring SNc dopamine neurons (Covey & Garris, 2009; Garris et al., 1997), SNL stimulation failed to evoke any dopamine release in the dorsolateral striatum, despite sharing a common conduit through the MFB.

The key finding here was that SNL stimulation evoked dopamine release exclusively in the tail striatum, which is consistent with studies describing this projection (Jiang & Kim, 2018; Menegas et al., 2015, 2018). Furthermore, SNc stimulation did not evoke dopamine release in the tail striatum, which was expected given there is no projection from the SNc to the tail striatum (Menegas et al., 2015, 2018). This projection exclusivity between adjacent populations of dopamine neurons is also observed between the SNc and VTA (de Jong et al., 2022). Interestingly, SNL dopamine neurons have other similarities with VTA dopamine neurons including the expression of VGlut2 suggesting they are capable of co‐release of glutamate (Poulin et al., 2018). Future studies are needed to investigate the possible co‐release of glutamate in the tail striatum by SNL dopamine neurons, like is seen in the ventral striatum by VTA dopamine neurons (Stuber et al., 2010; Zhang et al., 2015).

SNL‐evoked dopamine release in the tail striatum was not only smaller in amplitude but was also less reproducible than SNc‐evoked dopamine release in the dorsolateral striatum. There are several possible reasons for why this occurred. As previously discussed, dopamine neurons in the SNL are sparsely organized (Fu et al., 2012; González‐Hernández & Rodríguez, 2000), and stimulation might not always recruit a sufficient number of neurons to evoke detectable dopamine release. Secondly, the presence of a small separate population of GABAergic neurons located in the most dorsolateral part of the SNL (González‐Hernández & Rodríguez, 2000) could inhibit neighboring dopamine neurons preventing tail striatum dopamine release. By contrast, there are no such GABAergic neurons in the SNc (González‐Hernández & Rodríguez, 2000). Finally, while the activity of SNc dopamine neurons is regulated by D_2_ autoreceptors, these are not expressed in the SNL (Fu et al., 2012; Poulin et al., 2018, 2020) and this lack of autoregulation could lead to depolarization block in SNL dopamine neurons. Future studies specifically addressing the lack of D_2_ autoregulation on tail striatum dopamine release are required.

As described previously, stimulation of the MFB evoked dopamine release in the dorsolateral striatum consistent with other studies (Covey & Garris, 2009; Kuhr et al., 1987; Lloyd et al., 2022). We show here for the first time that such stimulation also evoked dopamine release in the tail striatum, indicating that both pathways project via the MFB. Dopamine release in the tail striatum was however less reproducible across experiments. Given that the tail striatum and SNL are located more caudally than the dorsolateral striatum and SNc, respectively, it is possible that the optimal location of stimulation along the MFB would be different for this dopamine pathway. Indeed, post hoc histological analysis revealed that dopamine release was only evoked in the tail striatum when stimulating at a more caudal site along the MFB.

### 4.3 Exclusive modulation of the novel dopamine pathway by the subthalamic nucleus

We showed that the STN has a unique role in the basal ganglia circuitry by exclusively modulating dopamine neurons of the SNL and evoking dopamine release in the tail striatum. This observation is consistent with a viral‐vector tracing study showing that tail striatum‐projecting dopamine neurons receive STN input (Menegas et al., 2015). While others have shown that STN stimulation evoked dopamine release in the *dorsolateral* striatum (Covey & Garris, 2009; Lee et al., 2006; Min et al., 2016), we were unable to replicate this observation. Given the proximity of the STN to the MFB, and the reported preference to evoke release in the dorsolateral striatum by stimulating the dorsomedial border between the STN and MFB, it is possible that inadvertent stimulation of the MFB could explain the evoked dopamine release they saw in the dorsolateral striatum. In the current study, we can be confident that the STN was specifically stimulated because STN stimulation never evoked dopamine release in the dorsolateral striatum even though MFB stimulation did so convincingly. Specific optogenetic stimulation of the STN would offer valuable insight into the selective modulation of distinct dopamine pathways and it is interesting to note that, despite the advantages of such an approach, no STN photo‐stimulated dopamine release in the dorsolateral striatum has been reported.

Furthermore, the anatomical and electrophysiological evidence shows that there is only a minor glutamatergic afferent projection from the STN to dopamine neurons in the SNc that would support STN‐evoked dopamine release in the dorsolateral striatum (Chang et al., 1984; Hammond et al., 1978; Kita & Kitai, 1987). However, there is a major STN glutamatergic projection to GABAergic neurons of the substantia nigra pars reticulata (SNr; Kita & Kitai, 1987; Parent & Hazrati, 1995), which in turn project to the SNc causing inhibition of these dopamine neurons (Paladini et al., 1999; Tepper & Lee, 2007). It is likely that this polysynaptic inhibitory mechanism (STN to SNr to SNc) may overpower the monosynaptic excitatory action of the STN on the SNc, resulting in no net dopamine release in the dorsolateral striatum, as we have observed. Detailed examination of the electrophysiological effects of STN stimulation on SNL in comparison with SNr and SNc neurons is yet to be done; an important knowledge gap given the pathophysiological hyperactivity of the STN in Parkinson's disease (Albin et al., 1989; Blandini et al., 2000), and the fact that this important nucleus is a target for deep brain stimulation therapy for the disease (Gill et al., 2011; Okun, 2012).

**FIGURE 6:**
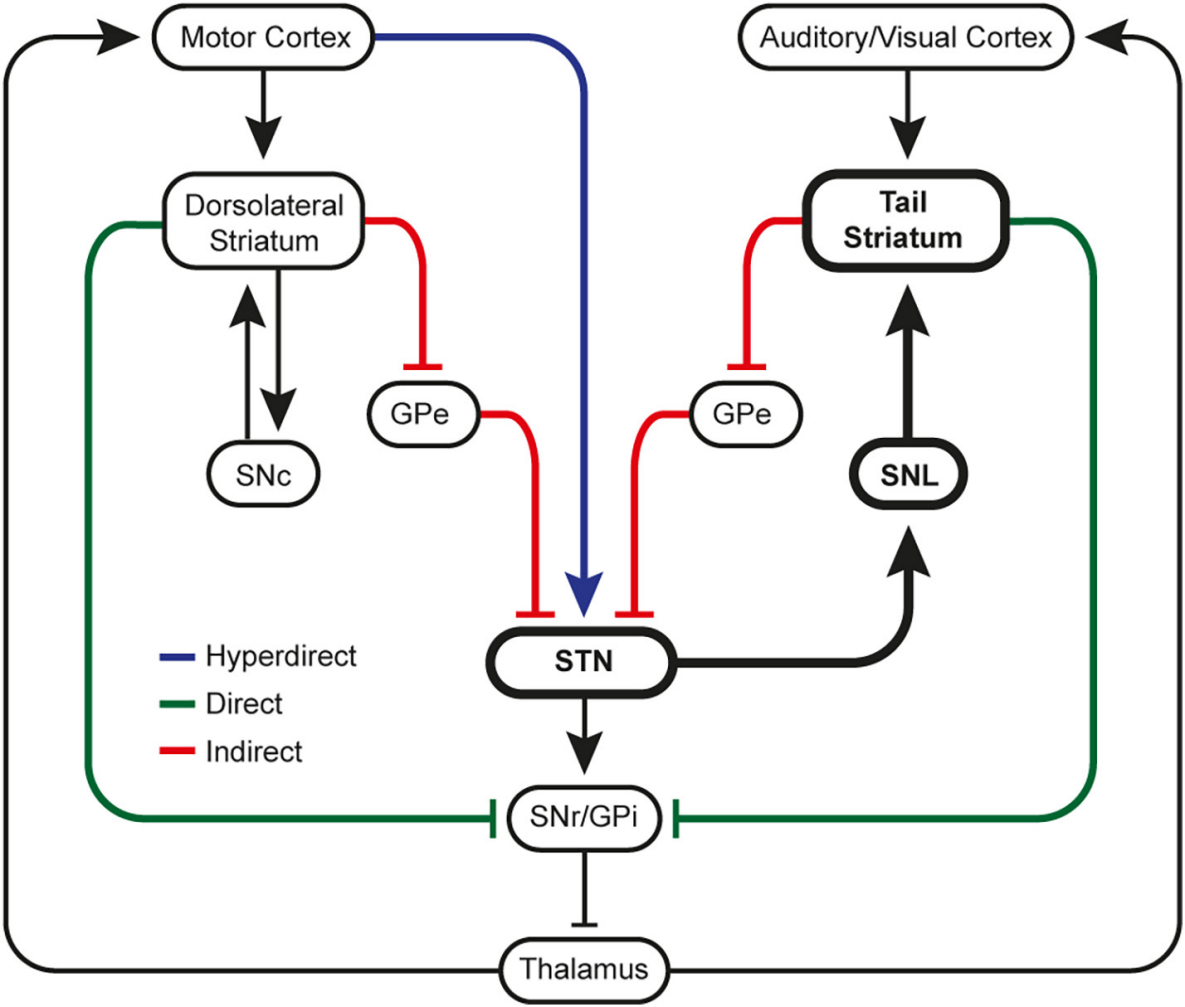
Proposed model of the basal ganglia. Cortical information from the motor cortex and auditory/visual cortex remains segregated in the dorsolateral and tail striatum dopamine domains, respectively. Information from the functionally distinct systems is integrated in the STN which receives direct motor input via the hyperdirect pathway and input from both striatal regions via the indirect pathway. The STN exclusively excites dopamine neurons in the SNL, which in turn modulates dopamine release in the tail striatum only (bold).

In light of our current finding that the STN exclusively modulates tail striatum‐projecting SNL dopamine neurons, and that the STN has a unique hyperdirect input from the motor cortex (Nambu et al., 2000, 2002) as well as input from the indirect pathway (via the GPe), we propose that the STN holds a unique position in the basal ganglia circuitry integrating motor and sensory information (Figure 6). Indeed, while information from the motor cortex and auditory/visual cortex remains segregated in the dorsolateral (sensorimotor domain) and tail (sensory domain) striatum, respectively (Hunnicutt et al., 2016), one key study showed that this cortical information converges in the STN (Kolomiets et al., 2001). Furthermore, within the STN a local excitatory network exists (Ammari et al., 2010; Shen & Johnson, 2006), which generates a higher level of convergence and integration of cortical information. The STN, via its unique projection to the SNL, therefore acts as the link between two parallel circuits allowing integration of information from functionally distinct cortical and striatal regions.

It is important to consider this revised basal ganglia circuitry in the context of Parkinson's Disease, especially given that in Parkinson's patients there is a greater reduction of dopamine in caudal portions of the putamen (Frost et al., 1993; Kish et al., 1988) and there is some evidence that SNL dopamine neurons are particularly vulnerable (Goto et al., 1989). This loss of the novel dopamine pathway could explain the non‐motor symptoms seen in Parkinson's patients which dominate early in the disease progression (Pont‐Sunyer et al., 2015) and are often untreated (Baig et al., 2015). Parkinson's patients often have difficulty in performing daily (habitual) routines (Redgrave et al., 2010) and show saccade anomalies (impairment in gaze orientation; Bakhtiari et al., 2020). Interestingly, dopamine neurons in the lateral SNc (SNL equivalent in primate) are involved in learning and sustaining habitual behaviors and contribute to saccade movements toward valuable objects (Kim et al., 2015). The STN has also been shown to be a key modulator of visuomotor action selection (Bakhtiari et al., 2020). These functional roles of the STN and SNL highlight the importance of the novel dopamine pathway and its modulation by the STN and how further research, especially in the context of Parkinson's disease, would be very valuable.

## AUTHOR CONTRIBUTIONS

P.S.F and J.L designed the research; K.L.T performed the research and analyzed the data; K.L.T and P.S.F wrote the paper; P.S.F and J.L reviewed and edited the paper.

## CONFLICT OF INTERESTS

The authors declare no competing interests.

### OPEN RESEARCH BADGES

This article has been awarded an Open Materials Badge because it provided all relevant information about the components of the research methodology needed to reproduce the reported procedure and analysis. More information about the Open Science Badges can be found at https://cos.io/our‐services/open‐science‐badges/

## Data Availability

The data that support the findings of this study are available from the corresponding author upon reasonable request.
